# Interplay between Selenium, selenoprotein genes, and oxidative stress in honey bee *Apis mellifera* L.

**DOI:** 10.1016/j.jinsphys.2019.103891

**Published:** 2019-06-07

**Authors:** Mohamed Alburaki, Kristina D. Smith, John Adamczyk, Shahid Karim

**Affiliations:** a The University of Southern Mississippi, Department of Cell and Molecular Biology, School of Biological, Environmental, and Earth Sciences, Hattiesburg, MS 39406, USA; b USDA-ARS Thad Cochran Horticultural Laboratory, Poplarville, MS 39470, USA

**Keywords:** Honey bee, Selenium, Oxidative stress, Selenoprotein-like genes, Antioxidant gene

## Abstract

The honey bee, *Apis mellifera* L., is a major pollinator insect that lacks novel “selenoprotein genes”, rendering it susceptible to elevated levels of Selenium (Se) occurring naturally in the environment. We investigated the effects of two inorganic forms of Se on biological traits, oxidative stress, and gene regulation. Using bioassay arenas in the laboratory, one-day old sister bees were fed *ad libitum* 4 different concentrations of selenate and selenite, two common inorganic forms of Se. The transcription levels of 4 honey bee antioxidant genes were evaluated, and three putative selenoprotein-like genes (SELENOT, SELENOK, SELENOF) were characterized as well as Sbp2, a Selenium binding protein required for the translation of selenoproteins mRNA. Oxidative stress and Se residues were subsequently quantified in honey bee bodies throughout the experiment.

Se induced higher oxidative stress in treated honey bees leading to a significantly elevated protein carbonyl content, particularly at the highest studied concentrations. Early upregulations of Spb2 and MsrA were identified at day 2 of the treatment while all genes except SELENOT were upregulated substantially at day 8 to alleviate the Se-induced oxidative stress levels. We determined that doses between 60 and 600 mg.Se.L^−1^ were acutely toxic to bees (< 48 h) while doses between 0.6 and 6 mg.Se.L^−1^ led to much lower mortality (7–16)%. Furthermore, when fed *ad libitum*, Se residue data indicated that bees tolerated accumulation up to 0.12 μg Se bee^−1^ for at least 8 days with a Se LC_50_ of ~6 mg/L, a field realistic concentration found in pollen of certain plants in a high Se soil environment.

## Graphical Abstract

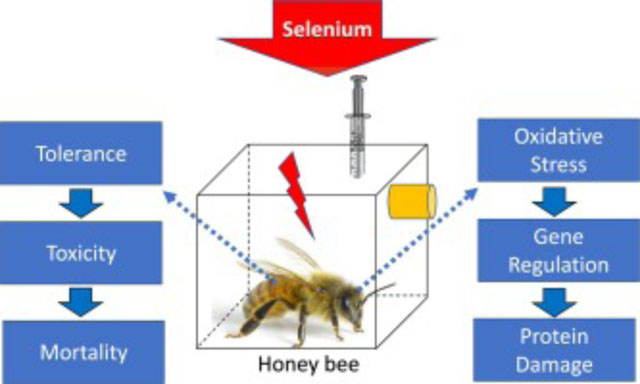

## 1. Introduction

Selenium (Se) is a nonmetal element that occurs naturally in some alkaline soils, plants and flowers. Se is a trace element nutrient for humans and other living organisms and functions as a cofactor for reduction of antioxidant enzymes, but it can be toxic at large doses. Se toxicity occurs due to the Se compounds’ reaction with glutathione (GSH) and other thiols forming selenotrisulfides that ultimately produce superoxide and hydrogen peroxide which are toxic for the organism (Chaudiere et al., 1992). Acute or chronic Se toxicity is manifested when the induced oxidative damage exceeds the organism’s antioxidant defenses or its ability to either produce selenoproteins or the complete absence of the latter (Spallholz, 1994). High levels of Se were described in the ecosystem of several western regions of the USA in poisoning fish, wildlife and livestock, causing deformation in birds and affecting pollinators (Marshall, 1986). Soil with high Se content, such as in Nebraska, Kansas, Dakotas, Wyoming (> 4–5 mg/kg), eventually results in high levels of Se plant uptake leading to toxicity in herbivores (Rosenfeld and Beath, 1964). Se in plants can be found in two forms: 1 – organic forms such as selenocystathionine and methylselenocystein and 2 – inorganic forms such as selenate and selenite (Freeman et al., 2006; Pickering et al., 2003; Underwood and Suttle, 1999). Elevated levels of Se in flowers, especially in Se-hyper-accumulating plants, can be toxic to many insect herbivores. Since there is no evidence that honey bees and other floral visitors can avoid high-Se flowers (Quinn et al., 2011), it is conceivable that the latter could put those economically important pollinators at great risk. The EPA maximum contaminant level of Se in drinking water is 0.05 mg/L and Se was identified in honey bees (*Apis mellifera* L.) at 14.8 μg/g and bumble bees (*Bombus* spp.) at 251 μg/g of their dry weight (Quinn et al., 2011). Honey bee foragers sampled from non-contaminated Se urban areas contained 0.73 mg Se kg^−1^. Se also was identified in forager bees in Poland at a concentration of 7.03 mg Se kg^−1^ dw (Roman, 2010).

Various levels of Se concentrations were described in honey as well. For instance, in Turkey, honey collected from various regions contained 0.038–0.113 mg Se kg^−1^ (Tuzen et al., 2007) and honey collected from hives located in seleniferous soils of Colorado (USA) contained approximately 0.73 mg Se kg^−1^ (Quinn et al., 2011). The bioaccumulation and biotransfer of Se from plants to pollinators, particularly in honey bees, as well as the tolerable doses of this element in bees are poorly known.

Selenoproteins have not been found in every living organism and their numbers vary significantly in mammals and are lost altogether in most arthropods. It has recently been demonstrated that a few insect species (*Bombyx mori*, *Drosophila willistoni*, *Nasonia vitripennis*), including the honey bee, *Apis mellifera*, a major pollinator insect, do not possess selenoproteins due to absence of the selenocysteine incorporation machinery (Hirosawa-Takamori et al., 2004; Shchedrina et al., 2011). Instead, those insect species and others possess cysteine-containing homologs, and further study of their selenoproteome revealed a significant reduction in the number of selenoproteins to only 1–3. This is the case, at least for *Drosophila melanogaster* and *Anopheles gambiae* (Shchedrina et al., 2011). Selenoproteins are involved in different biological processes and carry out multiple functions such as detoxification of peroxide, protein folding, repair of oxidative damage, regeneration of reduced thioredoxin and oxidation of the Selenium itself (Gromer et al., 2005; Labunskyy et al., 2014; Reeves and Hoffmann, 2009).

Honey bees possess very few selenoproteins which render them susceptible to Se (Lobanov et al., 2008), contrary to other organisms such as ticks, which generally have a sophisticated set of selenoproteins that play a major role in alleviating oxidative stress effects (Adamson et al., 2014; Budachetri and Karim, 2015; Budachetri et al., 2018).

Selenate (SeO_4_^−2^) and selenite (SeO_3_^−2^) have different biochemical properties and toxicities (Shen et al., 1997; Weiller et al., 2004) and are metabolized differently in both aquatic and terrestrial animals (Eisler, 2007; Somogyi et al., 2012, 2007). Both forms of inorganic Se, selenate and selenite, are known to cause damage to DNA at elevated concentrations, (Combs and Gray, 1998) and induce oxidative stress (Spallholz, 1997) in living organisms. Simple mortality may not be the only outcome of exposure to Se in honey bee colonies; several studies have shown that Se decreases the worker weight (Hladun et al., 2016) and has a sub-lethal effect on bee behaviors that are crucial to the survival of colonies (Burden et al., 2016).

Here, using laboratory bioassay arenas (i.e. cages), we studied the effects of selenate and selenite at various concentrations on honey bee biological traits and oxidative stress, as well as on regulation of major antioxidant genes. Furthermore, we investigated the activity of un-characterized genes annotated *in silico* as members of the selenoproteins group. We characterized the “selenoprotein genes” function vis-à-vis the exposure to Se and their involvement in the detoxification process including protein post-transcriptional damage.

## 2. Material & methods

### 2.1. Laboratory bioassay design

**Fig. 1.**
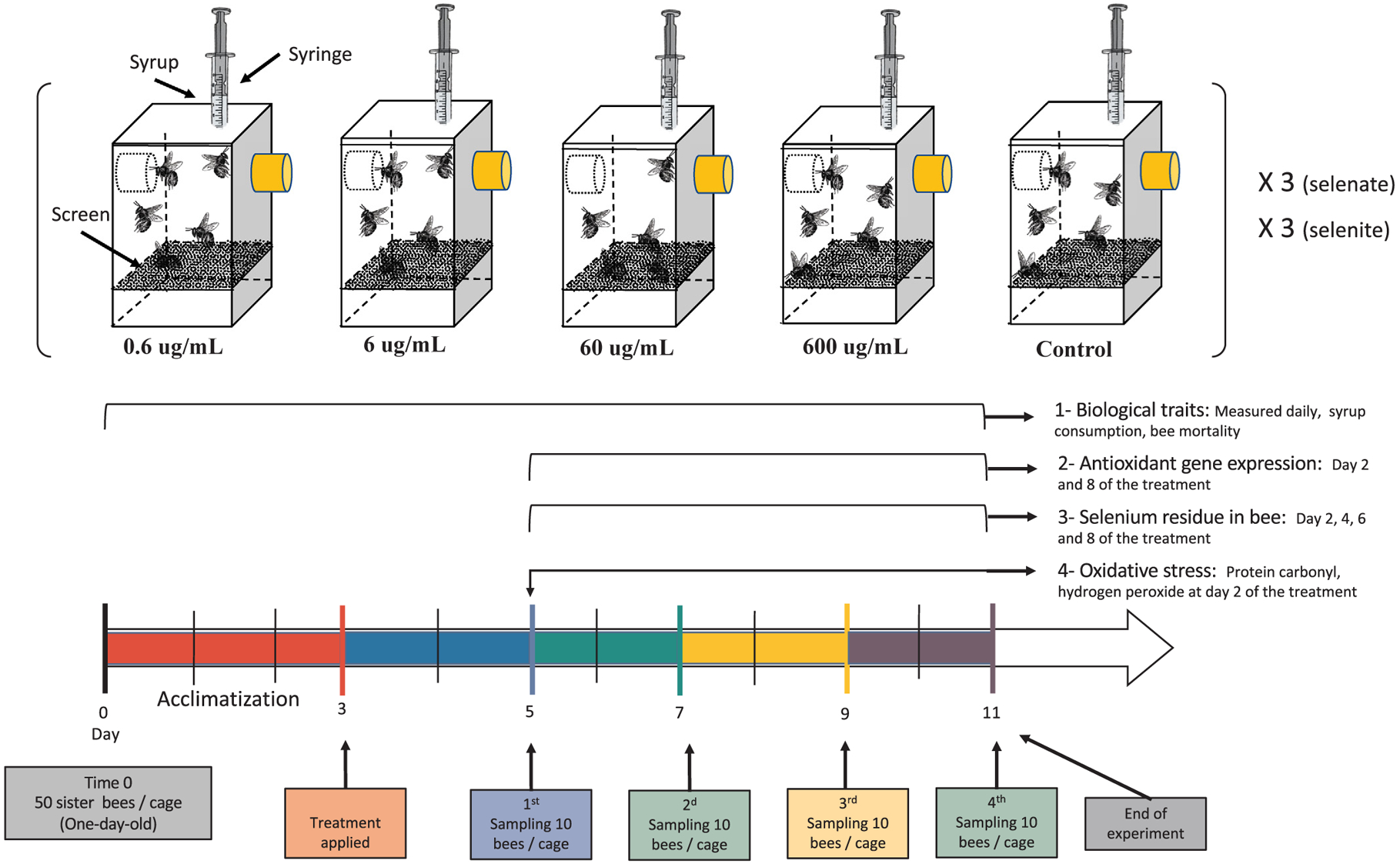
Shows the cage experimental design, concentrations of both selenate and selenite used in each treatment group as well as the sampling and acclimatization timelines. Each experiment lasted for 11 days and 50 sister bees were used per cage.

This experiment was conducted at the University of Southern Mississippi, Department of Cell and Molecular Biology. Two separate and similar cage experiments were conducted. Se was administrated to caged bees in two forms: sodium selenate (Na_2_SeO_4_, Sigma-Aldrich, Inc.) and sodium selenite (Na_2_SeO_3_, Amresco®, OH, USA), Fig. 1. Se was administrated to bees through 1 M tainted sugar syrup at various concentrations (0.6, 6, 60, 600) μg/mL and a control treatment with no Se, Fig. 1. These concentrations were chosen based on Se bee toxicity data of ECOTOX database (US Environmental Protection Agency EPA) and the very few available Se studies conducted previously on honey bees (Hladun et al., 2012; Quinn et al., 2011), which can offer comparison data. One-day old worker bees were hatched in the lab and distributed equally to 30 cages; 15 for each of the selenate and selenite experiments. These cages were specifically designed for feeding experiments (see Supplementary material) and are fully described in (Gregorc et al., 2018b). Each cage contained 50 worker bees and each treatment was conducted in triplicates, Fig. 1. Experiments lasted 11 days and consisted of two phases: 1 – 3-day-acclimatization period allowing bees to familiarize with cage conditions, 2 – a phase of 8-day-treatment. During the acclimatization period (prior treatment), all caged bees were fed 1 M sugar syrup with no applied treatment of any kind. Bees were subjected to their respective treatments from day 3 to 11 of the experiment, Fig. 1. Sugar syrup (1 M) was fed to bees *ad libitum* using 10 mL syringes as outlined in Fig. 1. The sugar syrup consumption was recorded daily from each syringe (0.2-mL sensitivity), as well as the number of dead bees. Dead bees were collected daily and stored at −80 °C for further chemical analysis. Four different samplings were performed from each cage at day (5, 7, 9, 11), which respectively corresponds to day (2, 4, 6, 8) from the starting treatment-day, Fig. 1. Ten bees per cage were sampled in each of those days and immediately stored at −80 °C for further chemical and molecular analyses.

### 2.2. Oxidative stress

#### 2.2.1. Hydrogen peroxide assay

This test was conducted to assess the honey bee physiological stress induced by exposure to Se. The level of H_2_O_2_ was quantified using the biological liquid of whole bee samples exposed to four selenate and selenite concentrations (0, 6, 60, 600) μg/mL. Bees were fed these concentrations *in vitro* through 1 M sugar syrup for 2 days while control bees were only administrated 1 M sugar syrup. Bees were individually crushed in 1.5 mL tubes with 300 μL ultra-sterilized water and centrifuged at 11,000g for 3 min. In order to eliminate the proteins, the supernatant containing the biological liquid was filtrated through a 10 kDa filter and the assay was conducted using BioVision Kit (CA, USA) as per the manufacturer’s instructions.

#### 2.2.2. Protein carbonyl content assay

To quantify potential post-transcriptional damage caused by Se exposure, we conducted a protein carbonyl assay. Proteins were solubilized from honey bee thorax in a protein extraction buffer consisting of 20 mM Tris-HCl pH 8.0, 30 mM NaCl, and 10% glycerol. The tissues were crushed by using a pestle and sonicated using a Bioruptor Pico (Diagenode) sonication device for 10 cycles of 30 s pulse and 30 s rest at 4 °C. Homogenates were centrifuged at 5000*g* for 10 min at 4 °C and the supernatants were collected. The protein carbonyl contents in studied samples were estimated using Sigma-Aldrich Kit (MO, USA) as described in the manufacturer’s protocol.

### 2.3. RNA extraction

RNA was extracted mainly from the whole bee body; in some cases, RNA was obtained from different bee tissues: head, thorax and abdomen to individually characterize the expression of the selenoprotein-like genes in those tissues. One out of the 10 worker bees sampled per cage was individually homogenized in liquid nitrogen and turned to powder. The homogenized product was quickly brushed into 1.5 mL sterilized tubes containing lyses buffer. All proceeding steps for RNA extraction were carried out in accordance with the manufacturer’s protocol (GE Healthcare, illustar™, RNAspin Mini Kit, Buckinghamshire, UK). RNA extractions were subsequently nano-dropped (Thermo Scientific NanoDrop ND 1000 Spectrophotometers) for RNA quantity and quality and were set at 500 ng and stored at −80 °C. In total, 6 bees per treatment were analyzed at day 2 and 8 of the exposure to Se.

### 2.4. Transcriptional analysis

The gene expression activity of eight antioxidant genes was evaluated for caged bees fed with selenate only. cDNA was built from RNA extractions using BioRad iScript Kit following the manufacturer’s protocol. Primers were designed for both target and housekeeping genes using Primer 3 software available online on NCBI website, Tables 1 and 2. To confirm the expression of the three selenoprotein-like genes and Sbp2, we conducted a RT-PCR on a representative set of treated (6 μg/mL) and untreated samples and ran an electrophoresis on 1.5% agarose gel. PCR products were subsequently sequenced and amplified fragments were blasted against their respective target genes for confirmation. All RT-qPCR runs were based on 3 biological and technical replicates per sample, and were conducted on the treatment group of the highest Se concentration that provided the longest bee survivorship (6 μg/mL), enabling a greater longitudinal analysis. Target genes were normalized against two housekeeping genes (GAPDH, RPS18) known for their stability in honey bee tissues (Alburaki et al., 2017; Scharlaken et al., 2008), Table 2.

### 2.5. Quantification of Selenium residue

Two bees were randomly selected for Se residue quantification from each sampling date and cage. Bees were homogenized in a single 1.5 mL tube with 600 μL PBS buffer and kept at RT overnight. Samples were vortexed and sonication was performed for 10 cycles 30 sec each using a bioruptor Pico at 4 °C. Samples were vortexed again and 200 μL of the liquid phase was transferred to a fresh 1.5 mL tube and a total of 95 samples including the dead bees collected daily from cages were analyzed. Inductively coupled plasma-mass spectroscopy (ICP-MS) was utilized to quantify low concentrations of Selenium from the bee tissues as previously described (Budachetri et al., 2017). Briefly, tissues were analyzed with a sector field mass spectrometer (ThermoFisher Element XR) in high-resolution mode. Isotope scanning used 115ln as an internal standard, while 77Se and 78Se were detected. Device calibration was conducted using a set of external standards and PBS buffer was used as a negative control. The Se limit of detection LOD was (0.01–0.02) μg/g.

### 2.6. Statistical analysis

The cage experiment of this study was based on 3 biological replicates for each treatment and 3 technical replicates for each of the qPCR runs. To assess potential inter-cage/biological replicate variability, all transcriptional analysis was conducted at the individual bee level while Se residue analysis was based on a pool of 2 bees per analyzed sample as described in the material and methods section. Oxidative stress analyses were conducted at bee level on 6 biological replicates for each treatment. All figures and statistical analysis related to the biological traits, including Se residues in honey bee bodies, were generated and performed in the R environment (R Core Team, 2011). The main R libraries used for this task were “ggplot2”, “doBy” and “plyr”. All error bars of this study represent the standard error SE except for the boxplots (Box-and-whisker plots) in which datasets were represented by their quartiles and outliers. Gene expression was quantified in a quantification relative to the control and normalized against 2 housekeeping genes as described in the material and methods section. The transcriptional analysis and figures including the gene studies were carried out using Bio-Rad CFX Maestro™ Software, Version 1.1. Analysis of Variance ANOVA was conducted across the study at 95% confidence interval with the three standard levels of significance: *P < 0.05, **P < 0.01 and ***P < 0.001.

## 3. Results

### 3.1. Syrup consumption and bee mortality

**Fig. 2.**
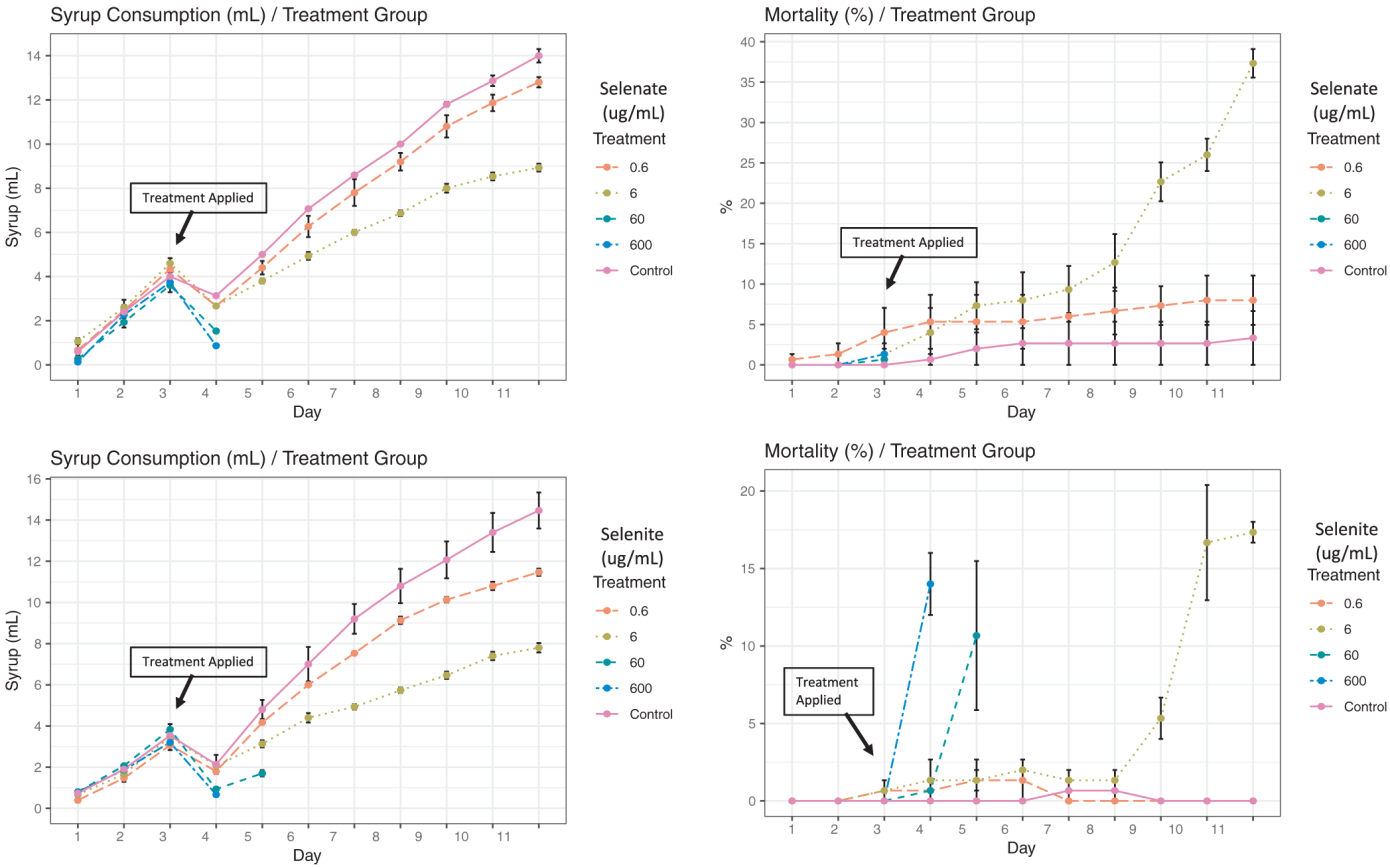
Syrup consumption throughout time exposed by treatment groups for both administrated selenate and selenite. Percentage of the daily bee mortality is also given for each treatment group. Day 1–3 is an acclimatization period where no treatment was applied. Treatments were applied at day 3 and lasted until day 11. Each data point represents (means ± SE) of 3 biological replicates.

**Fig. 3.**
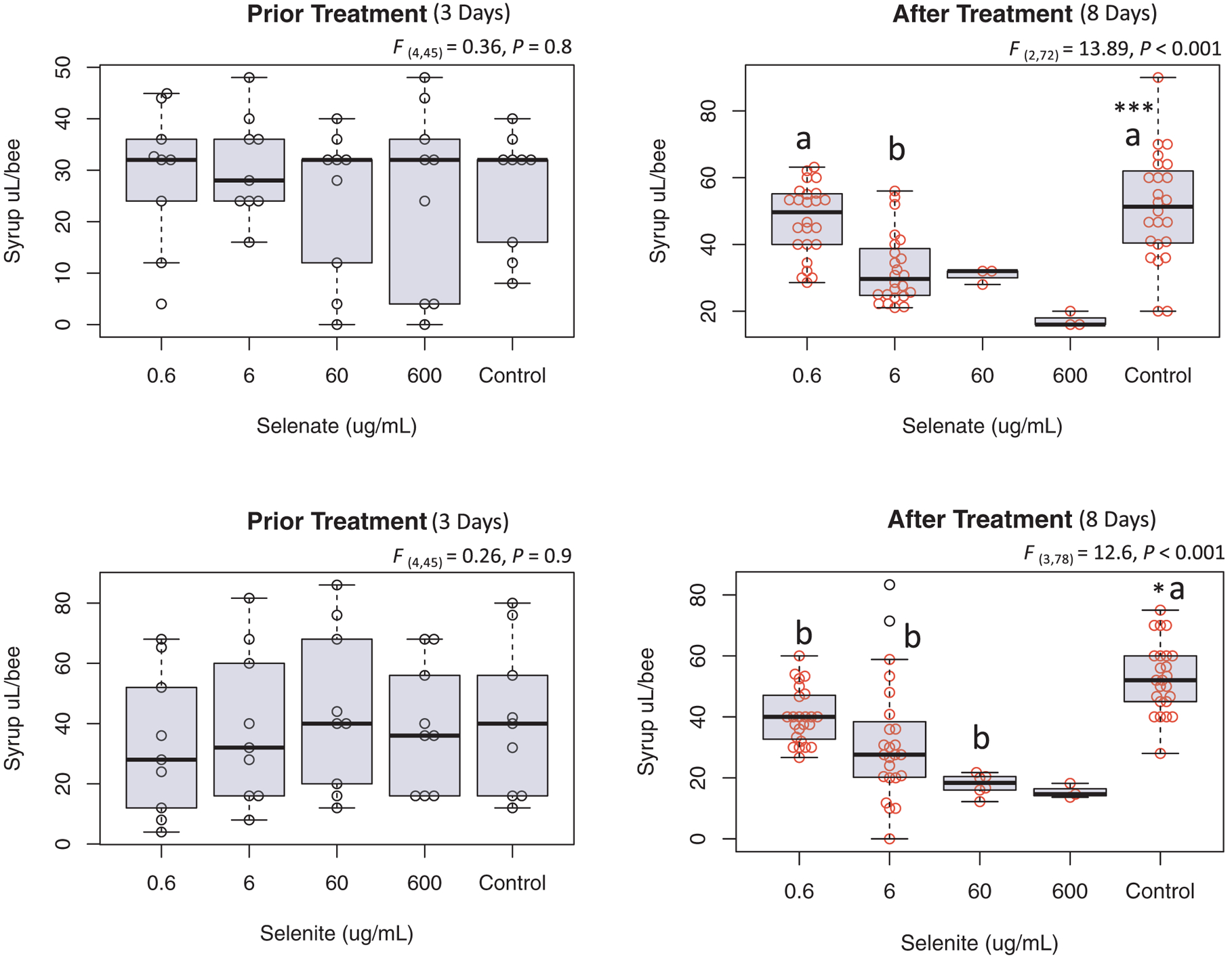
Average of syrup consumption per bee. Average syrup consumption for each treatment group prior and after treatment for both selenate and selenite. Prior treatment represents the acclimatization period with no treatment applied while post treatment is the consumption from day 3 and onward. ANOVA levels of significance are *P < 0.05 and ***P < 0.001. Inter-group significant differences are indicated with different letters.

**Fig. 4.**
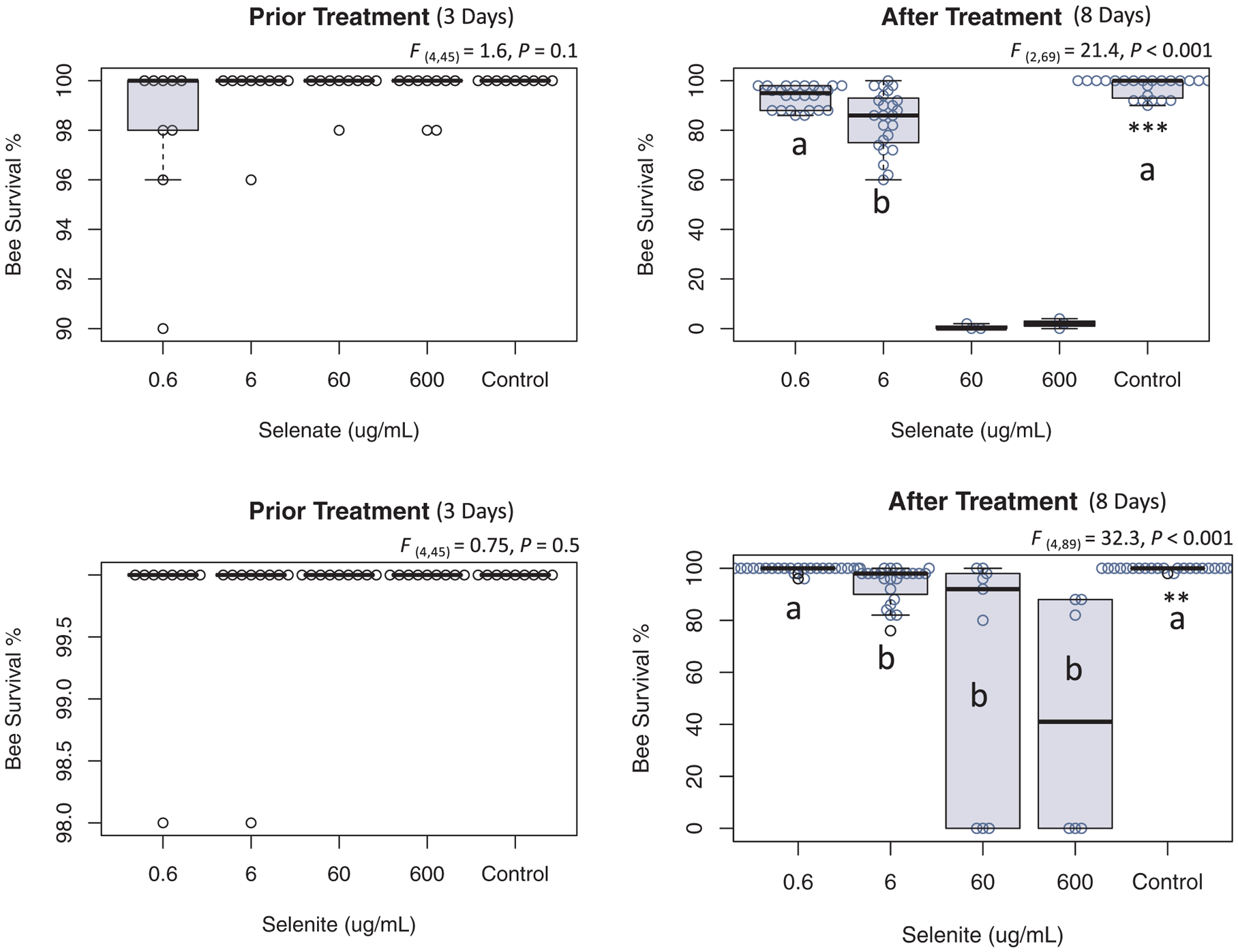
Average of bee survivorship. Percentage of bee survival for each treatment group for both selenate and selenite. Prior treatment stands for the acclimatization period in which all groups were only fed sugar syrup with no treatment. After treatment indicates the day 3 when treatments were administrated to bees and onward. ANOVA levels of significance are **P < 0.01 and ***P < 0.001. Inter-group significant differences are indicated with different letters.

During the acclimatization period (Fig. 1; day 1–3), bees of all treatment groups consumed equal amounts of syrup with no significant variation recorded (Figs. 2 and 3). Similarly, bee mortality showed no differences (P = 0.1 and 0.5) among groups prior to treatment at any time point, Fig. 4. Differences in the syrup consumption started to vary significantly right after applying the treatment of both Se forms. The highest concentrations of selenite (60 and 600) μg/mL, exhibited acute toxicity to honey bees killing all caged bees within ~24 h, Fig. 4. Due to bee mortality, post treatment daily average consumption with less than six reads was not included in the ANOVA, Fig. 3. Caged bees that were fed both of the other concentrations (0.6 and 6) μg/mL survived throughout the experiment and consumed significantly (P < 0.001) less syrup at 6 μg/mL compared to the control, with no differences between the control and 0.6 μg/mL, Fig. 3.

Post treatment syrup consumption for selenite showed significantly higher syrup consumption (P < 0.05) in the control compared to treated groups, Fig. 3. Selenite induced higher bee mortality at all concentrations compared to the control (P < 0.01) except for the lowest selenite concentration group (0.6 μg/mL), which did not significantly differ from the control, Fig. 4. Note that the overall survivorship of (60 and 600) μg/mL should be set at 0%, but we kept their reads to detail the fact that bees survived slightly longer (~48 h) than the bees exposed to similar concentrations of selenate, Fig. 4.

### 3.2. Honey bee oxidative stress

**Fig. 5.**
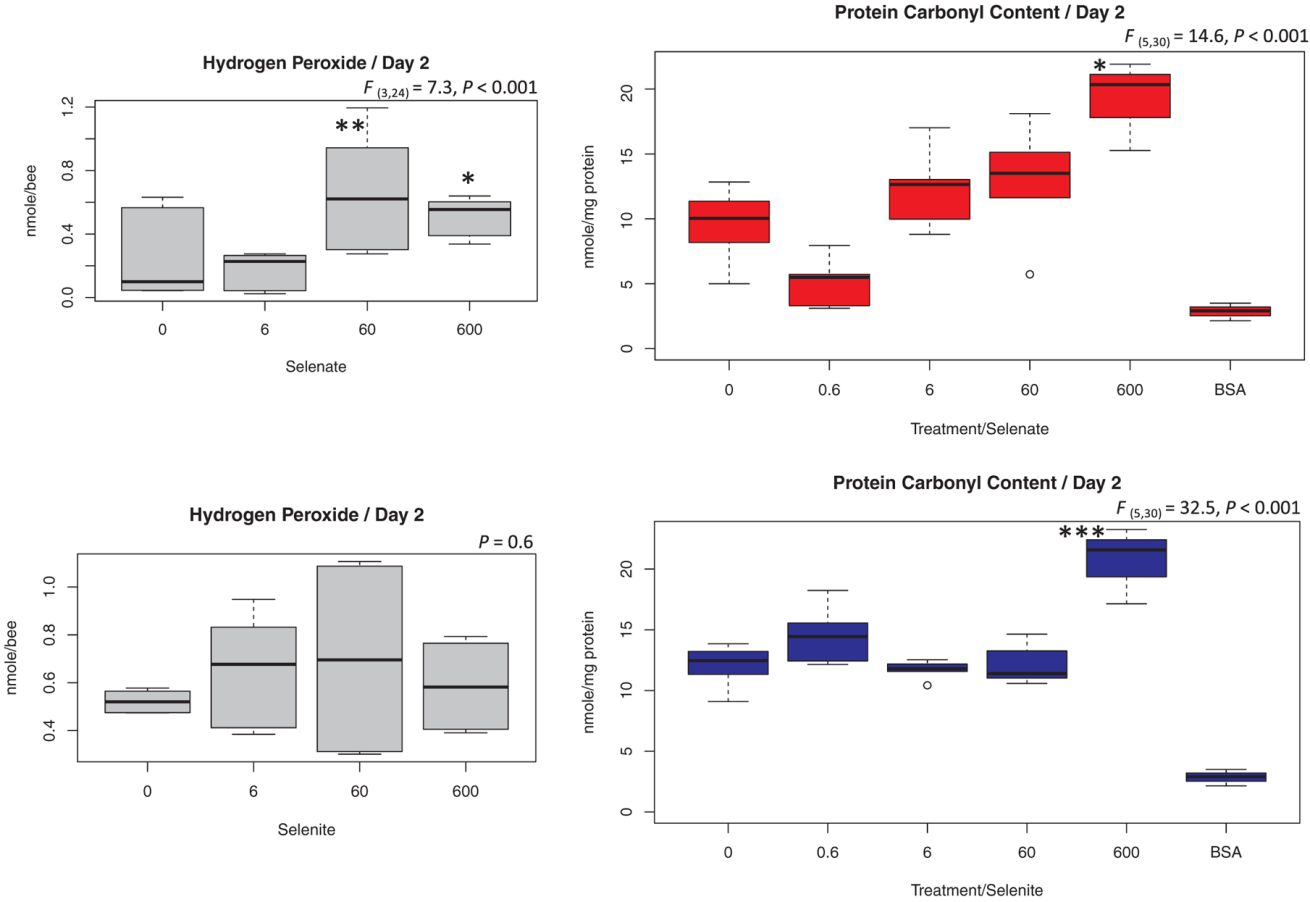
Honey bee Oxidative Stress. Quantification of both hydrogen peroxide and protein carbonyl content in honey bees fed various concentrations of selenate and selenite at days 2. All treatment concentrations are in (μg/mL) and BSA is bovine serum albumin. Each boxplot represents an average of 6 biological replicates and BSA was not included in the statistical analysis. Error bars are the quartiles and outliers and ANOVA levels of significance are *P < 0.05, **P < 0.01 and ***P < 0.001.

Both hydrogen peroxide and protein carbonyl contents were measured at day 2 of the exposure to Se. Bees fed (60 and 600) μg/mL selenate contained significantly higher hydrogen peroxide than other groups, while no significant differences were found among selenite-fed bees, Figs. 5 and 11. However, the protein carbonyl content was exclusively higher in the 600 μg/mL group for both selenate (P > 0.05) and selenite (P < 0.001), Fig. 5.

### 3.3. Transcription of selenoprotein-like gene

**Fig. 6.**
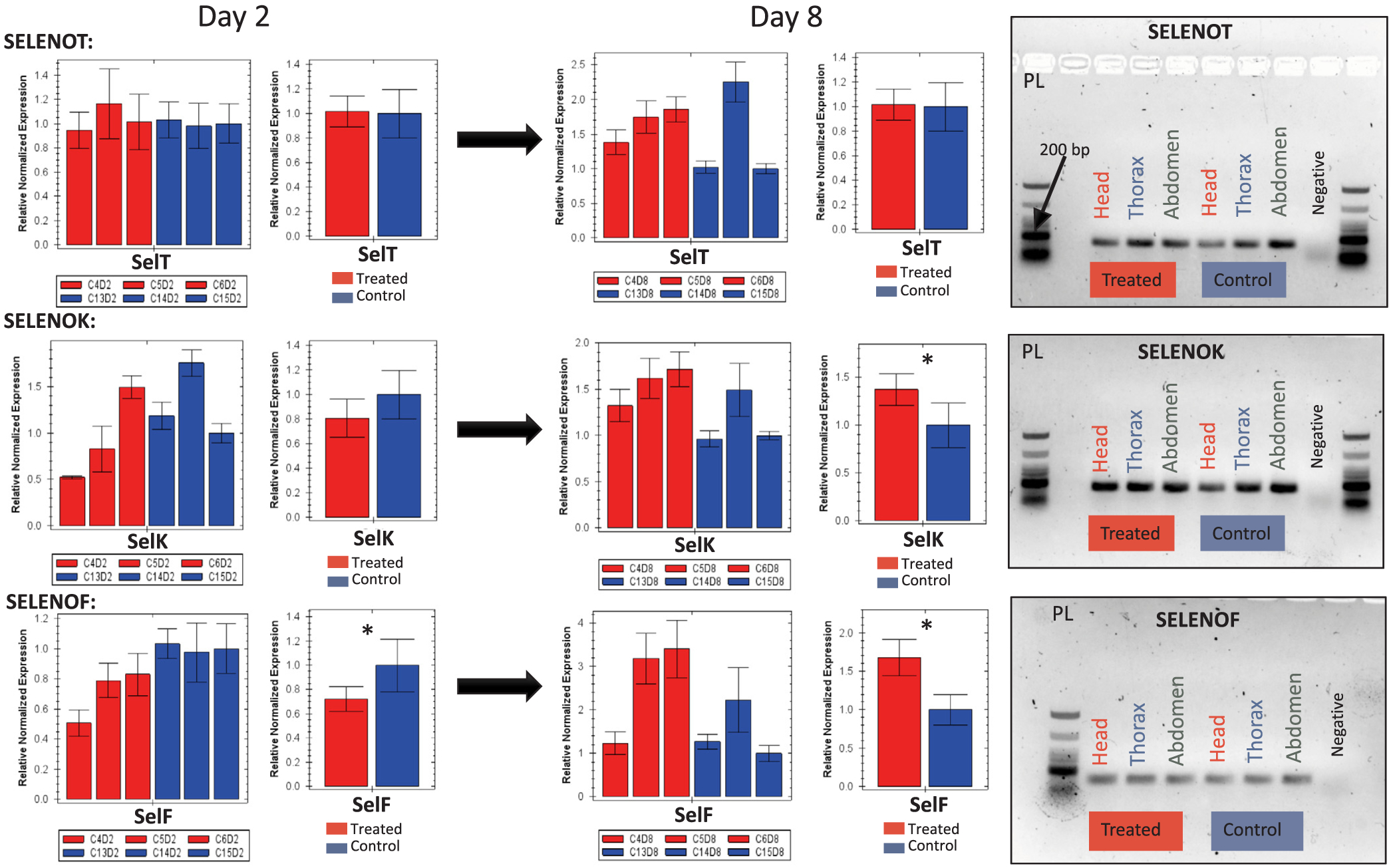
Relative quantification conducted by RT-qPCR of three selenoprotein-like genes (SELENOT, SELENOK, SELENOF) exposed by individual biological replicates (cage) and total average (means ± SE) of 3 biological replicates for both selenate fed bees (6 μg/mL) and control bees. Gene expression is studied at two different time points (day 2–day 8) of the experiment. The 1.5% agarose gels show inter-tissue transcriptional verification of studied genes conducted by RT-PCR for representative set of samples (6 μg/mL selenate vs. control). Error bars represent the Standard Error SE and level of significance is *P < 0.05.

**Fig. 7.**
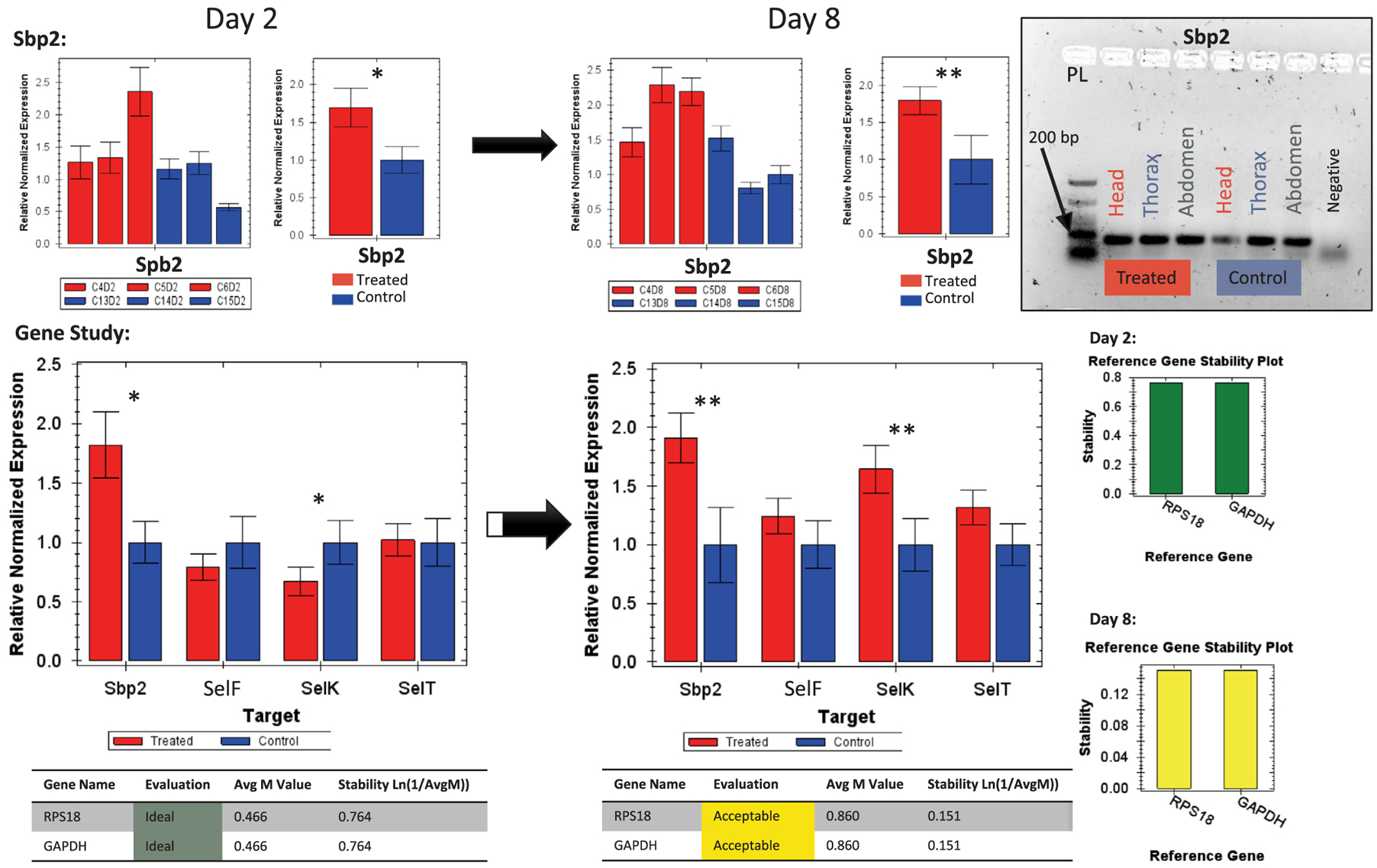
Relative quantification of the Selenium binding protein gene (Sbp2) at day 2 and 8 of the treatment conducted by RT-qPCR. The gene study describes the four target genes normalized against two housekeeping genes (GAPDH and RPS18). Sbp2 inter-tissue transcriptional verification run on 1.5% agarose gel is also shown. Error bars are the Standard Error SE and levels of significance are *P < 0.05, **P < 0.01. Housekeeping gene stability is given for each gene.

Regardless of the Se treatment, RT-PCR results of the selenoprotein-like and Selenium binding protein genes (SELENOF, SELENOK SELENOT, Sbp2) clearly indicate active expressions of those genes in all bee tissues (head, thorax and abdomen) with more band intensity in the treated bees for some genes, Figs. 6 and 7.

### 3.4. Gene regulation

The majority of the eight antioxidant genes (Tables 1 and 2) evaluated in this study showed interesting regulation vis-à-vis Se administrated orally to bees from day 2 to day 8 of the exposure, Figs. 7 and 9. SELENOT was the only gene that exhibited no regulation throughout time, while SELENOK upregulated (P < 0.05) at day 8, Fig. 6. Interestingly, SELENOF switched from downregulation at day 2 to upregulation at day 8 (P < 0.05) always compared to the control bees, Fig. 6. However, an increase of upregulation was recorded for Spb2 from day 2 (P < 0.05) to day 8 (P < 0.01).

When accounting all the selenoprotein-like genes together in a single gene study normalized against both GAPDH and RPS18 housekeeping genes, two genes were revealed to have the main expression in the dataset, Spb2 and SELENOK. Sbp2, already upregulated at day 2, intensified its regulation at day 8 while SELENOK, which was down-regulated (P < 0.05) at day 2, significantly upregulated (P < 0.01) at day 8, Figs. 7 and 11.

**Fig. 8.**
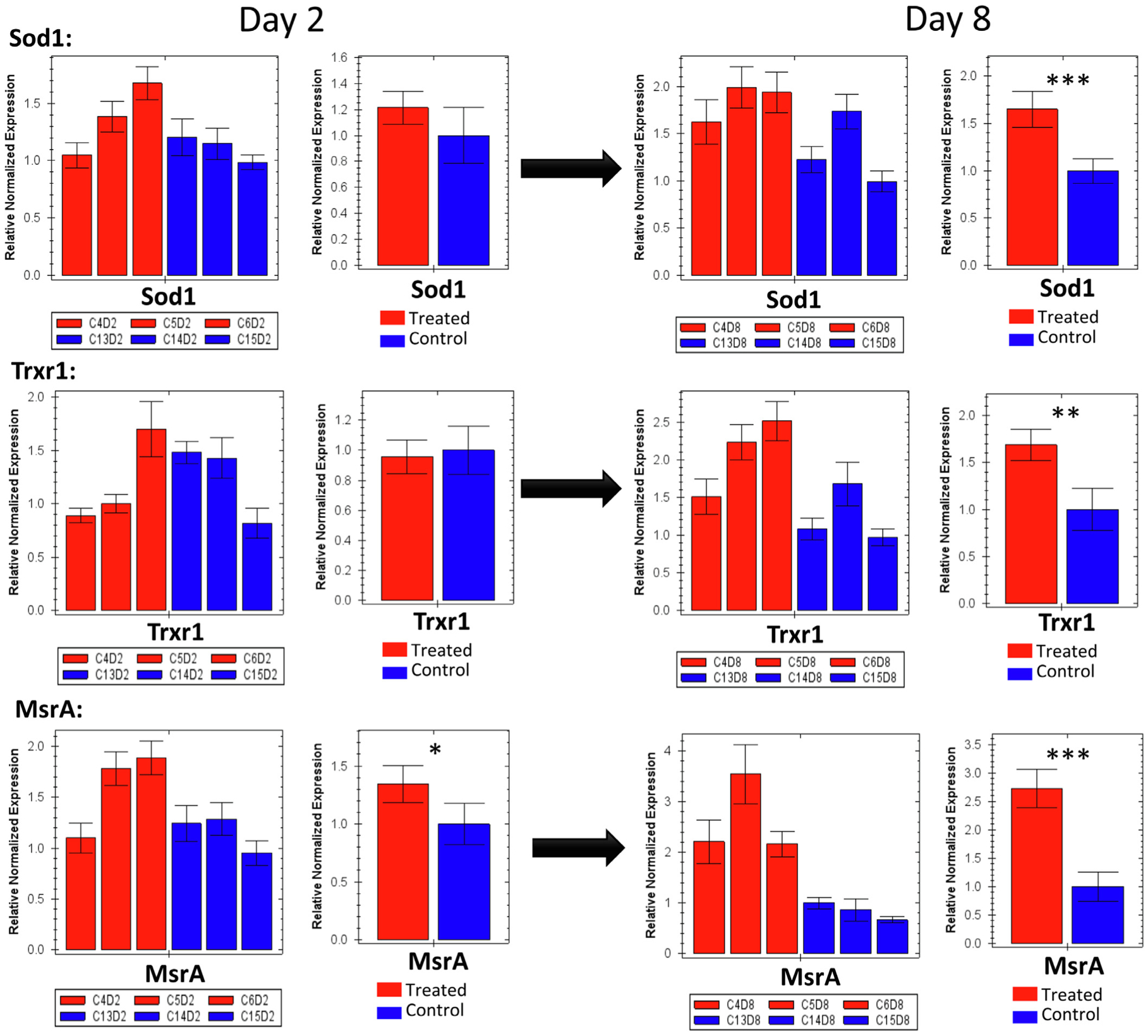
Relative quantification conducted by RT-qPCR of major honey bee antioxidant genes (Sod1, Trxr1, MsrA) exposed by individual biological replicates (cage) and total average (means ± SE) of 3 biological replicates for both selenate fed bees (6 μg/mL) and control bees. Gene expression is studied throughout time from day 2 to day 8 of the treatment and conducted on samples of the same cages (6 μg/mL vs. Control). Error bars represent the Standard Error SE and levels of significance are *P < 0.05, **P < 0.01, ***P < 0.001.

**Fig. 9.**
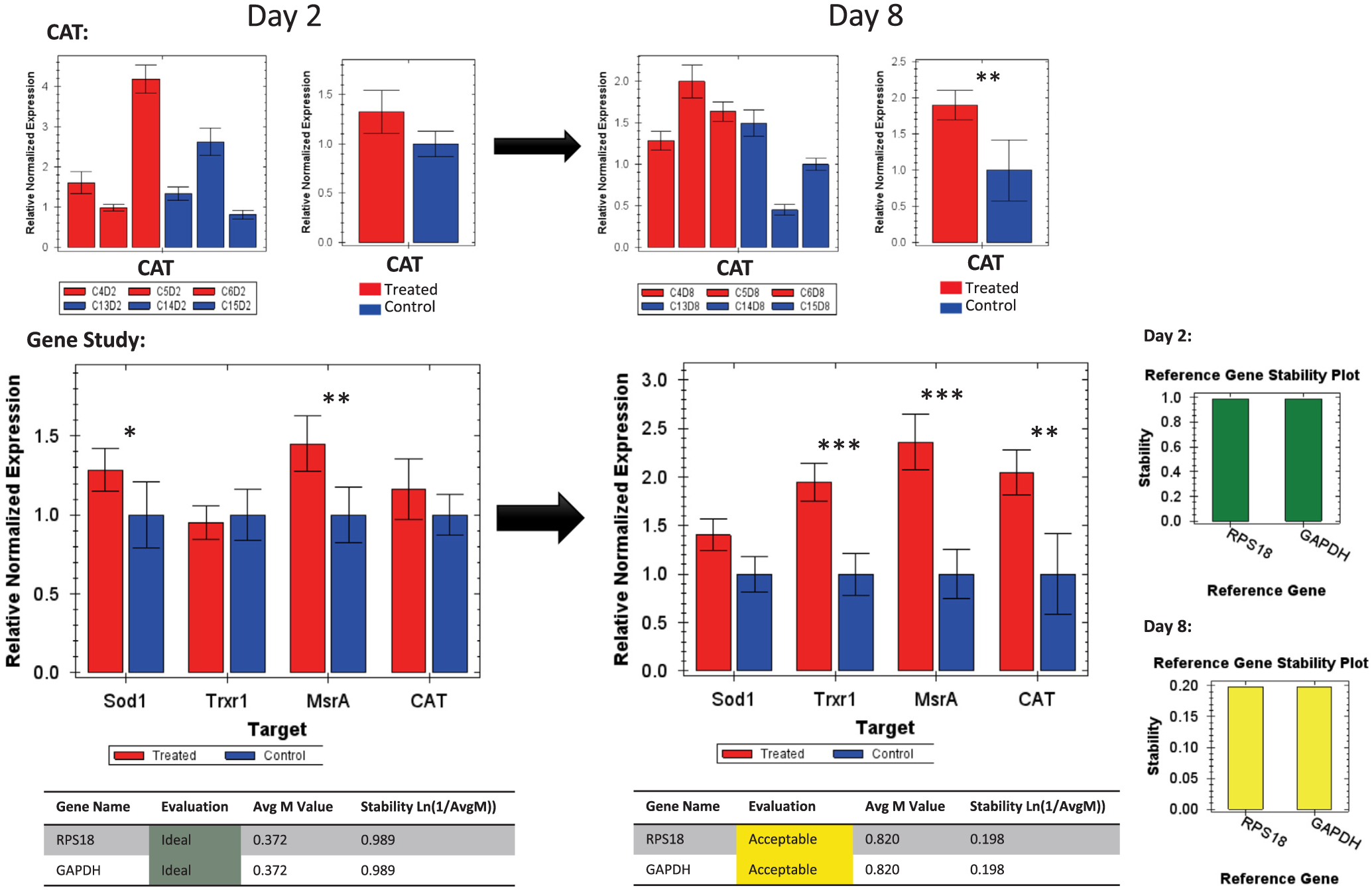
Relative quantification of the catalase (Cat) at day 2 and 8 of the treatment conducted by RT-qPCR. Gene study of the four target genes normalized against two housekeeping genes (GAPDH and RPS18). Error bars are the Standard Error SE and levels of significance are *P < 0.05, **P < 0.01, ***P < 0.001. Housekeeping gene stability is given for each gene.

Concerning the regulation of the four major honey bee antioxidant genes (Sod1, Trxr1, MsrA, Cat), all of those genes significantly upregulated with no exception at the last day of the exposure to Se (day 8), Figs. 8 and 11. When considering the four genes’ activities together, the gene study concluded similar results with the exception of Sod1 that was qualified as non-regulated at day 8, Fig. 9.

### 3.5. Honey bee Selenium residue

**Fig. 10.**
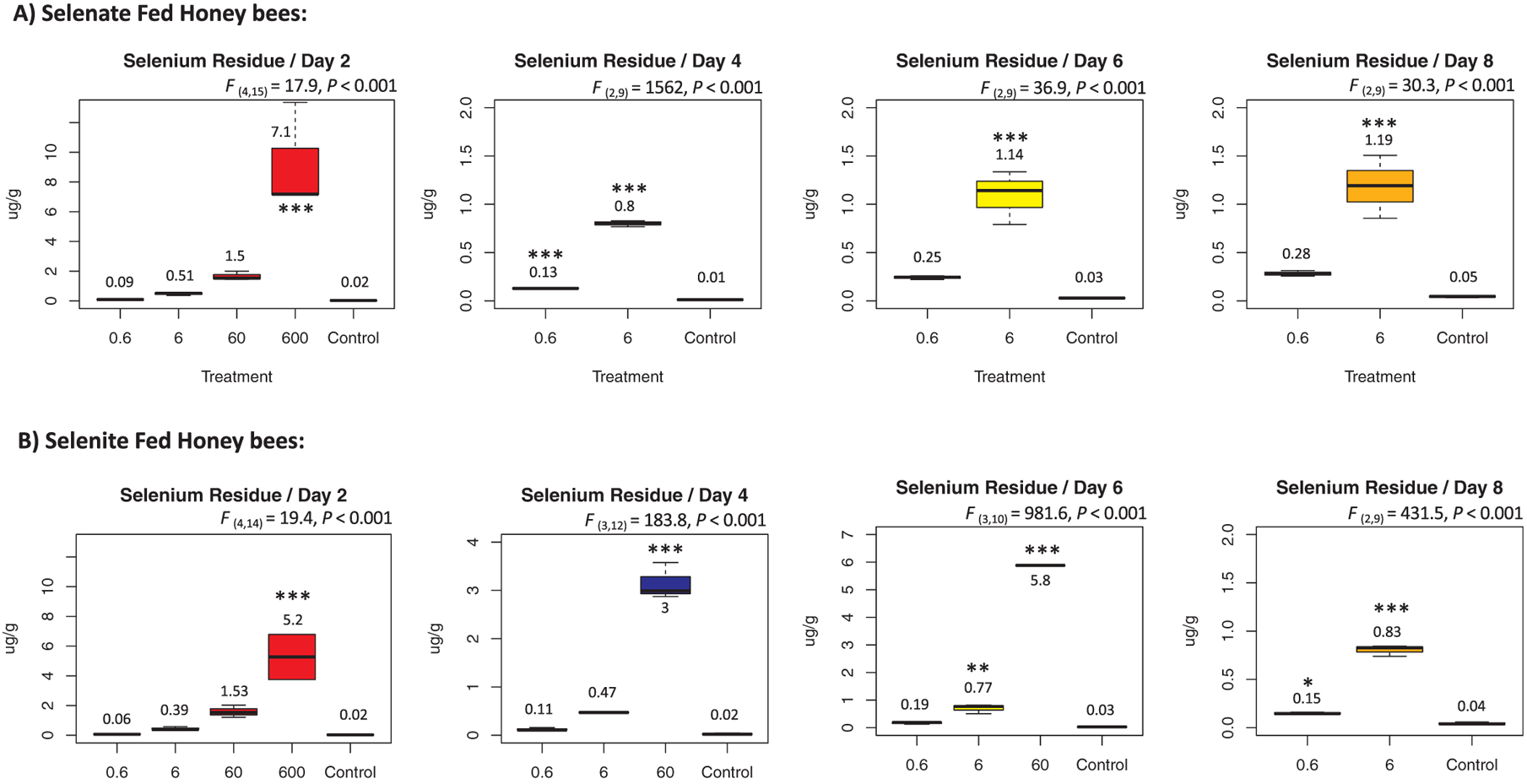
Selenium residues (μg/g) identified in each of the four sampled sets of bees at day 2, 4, 6 and 8. Results are exposed by treatment groups and control for both selenate (A) and selenite (B) fed bees. Each boxplot represents an average of 3 biological replicates and error bars describe the quartiles and outliers. ANOVA level of significance among groups is ***P < 0.001.

Se residues for both selenate and selenite in bees of the control cages were constantly close to the LOD throughout time ranging between (0.01 and 0.05) μg/g, indicating absence of Se in the control bees, Fig. 10. The highest concentrations of Se residues were recorded at day 2 for the (600 μg/mL) treatment group, in which Se was identified at 7.1 and 5.2 μg/g respectively for both selenate and selenite fed bees, Fig. 10a & b. Both concentrations were revealed to be acutely lethal as bees of this group survived for only 24 h in the best case, Fig. 2. The accumulative residue of Se in bees fed 8 days on 0.6 μg/mL selenate ranged from (0.09 to 0.28) μg/g and (0.51 to 1.19) μg/g for those fed 6 μg/mL. For selenite, Se residue range for the 0.6 μg/mL group was (0.06–0.15) μg/g and (0.39–0.83) μg/g for the 6 μg/mL group, Fig. 10. Concerning the Se residue in the dead bees, it ranged from 0.1 to 7.03 μg/g, Table 3.

## 4. Discussion

The two inorganic forms of Selenium tested in this study were found to be acutely toxic to honey bees at concentrations between 60 and 600 μg/mL. A comparative toxicity study of selenate and selenite to amphipod *Hyalella azteca* indicated that selenite is 2–4 times more toxic than selenate (Brasher and Ogle, 1993). However, in certain soil organisms such as the potworm, *Enchytraeus albidus* (Somogyi et al., 2007), and larvae of the fly, *Megaselia scalaris,* (Jensen et al., 2005), selenate was found to be more toxic than selenite.

**Fig. 11.**
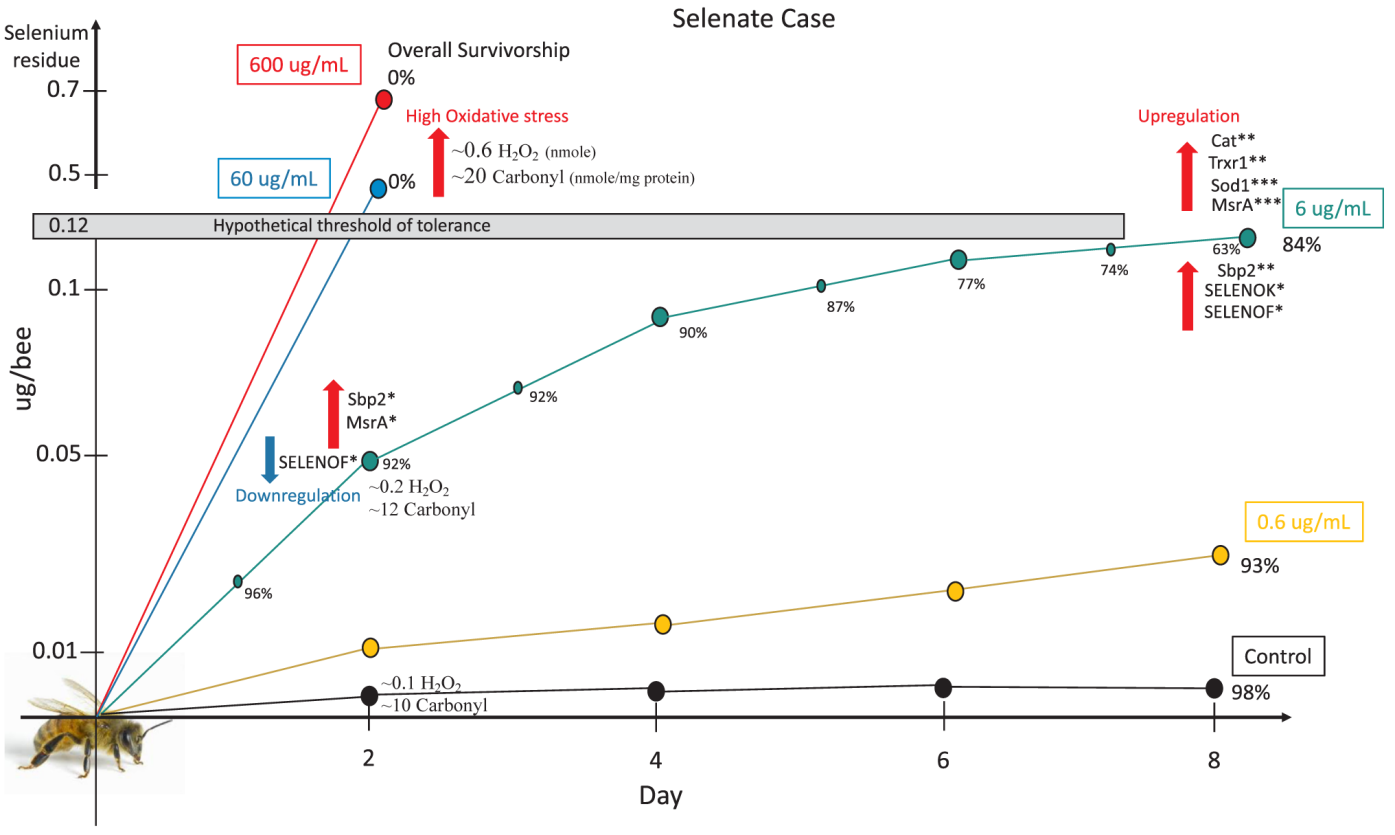
Schema of the selenate case summarizing the major transcription and post-transcription events related to the Selenium residue levels identified per bee in each of the treatment groups. Overall percentage of survivorship is given for each treatment group as well as daily survivorship percentages for the (6 μg/mL) group.

The third highest concentration of Se administrated to honey bees in this study (6 μg/mL) is the most telling and interesting concentration. Bees survived this concentration throughout the duration of the experiment (8 days), with an overall survivorship of 84%, significantly lower than the control (98%) and the 0.6 μg/mL treatment group (93%), Figs. 4 and 11. The divergence in the syrup consumption between the control and treated bees recorded in our study (Figs. 2 and 3) is not attributed to the bees’ ability to sense the presence of the Se (Hladun et al., 2012), but rather to the discomfort and illness caused to bees by the previous dose which prevents them from further abundant consumption of the treated syrup. This post-ingestive aversion response is developed by a process involving associative learning and was previously described in invertebrate herbivores and honey bees (Behmer et al., 2005; Meikle et al., 2016).

Exposure to Se led to significant increases in honey bee oxidative stress, reflected by a higher hydrogen peroxide content in treated bees compared to the control, Fig. 5. Inorganic forms of Se were previously identified as oxidative stress inducers (Spallholz, 1997). The elevated levels of hydrogen peroxide occurred as a response to ROS activity induced by the exposure to Se, which if not effaced by a detoxification process could lead to bee death or protein damage. We, indeed, identified significantly higher protein carbonyl contents (~20 nmol/mg protein) in bees given the highest concentration (600 μg/mL), which points to severe cellular damage taking place, Fig. 5.

In a previous study, the early selenate concentration causing significant bee mortality compared to the control was found to be > 600 μg/mL for both single and chronic selenate doses (Hladun et al., 2012). However, our data showed that both selenate concentrations (60 and 600) μg/mL were acutely lethal, killing all bees within 24 h, while early signs of significant bee mortality compared to the control were found in the 6 μg/mL treatment group. A previous study reported Se LC_50_ values of (1 and 58) μg/mL for larvae and adult bees respectively (Hladun et al., 2013), which is higher than what was found in our current study in the case of adult bees. Hladun et al. used forager bees in their study, which could explain the difference in honey bee toxicity levels obtained in younger bees in this current study. Living bees exposed to (6 μg/mL) contained 0.12 μg Se bee^−1^ at day 8 of our experiment (Fig. 10), which led us to identify a honey bee tolerable Se threshold of 0.12 μg Se bee^−1^, Fig. 11. It is however, surprising that Quinn et al. (2011) identified such high levels of Se (1.3–1.5) μg Se bee^−1^ in living honey bees foraging in high-Se flowers, Table 3. According to the same authors, those identified Se concentrations could have been derived from both forager bee bodies and the carried pollen.

Our transcriptional data conducted on bees of the 6 μg/mL group showed that in order for bees to alleviate the previously described oxidative stress, they significantly upregulated the majority of their antioxidant genes (Cat, Sod1, Trxr1, MsrA) at day 8, including the “selenoprotein genes”, Figs. 8 and 9. The intensive gene upregulation recorded at day 8 highlights the active process to alleviate the Se-induced oxidative stress in treated bees, Fig. 11. The Se residues identi-fied in bees of this group is particularly interesting and supports the gene regulation process. For instance, at day 2, bees contained only 0.051 μg Se bee^−1^ which hypothetically required upregulation of fewer genes (Sbp2, MsrA) than day 8, in which Se amount doubled (0.11 μg Se bee^−1^) leading to a full antioxidative process display, Figs. 9 and 11.

Our data shed more light on the functional role of the honey bee “selenoprotein-like genes” and Spb2 annotated *in silico* by the NCBI Eukaryotic Genome Annotation Pipeline. It is clear from the RT-PCR result that all four genes are translated and expressed across various bee tissues and extensively upregulated when bees are exposed to Se with the exception of SELENOT, which showed no regulation at any time point, Figs. 6 and 7. The early and constant upregulation of Spb2 is particularly interesting in the case of Se and still needs more investigation. It is worth mentioning that we recorded similar upregulation for Sbp2 and (CAT and Trxr1) in our previous study testing the effect of imidacloprid on honey bee gene regulation (Gregorc et al., 2018a). The automatic annotation of selenoproteins remains challenging due to the dual role of the termination signal codon (UGA), which also codes for the 21st amino acid, selenocysteine (Sec) (Castellano et al., 2005). Thus, it is conceivable that the automatic annotation of those genes as encoding for selenoproteins lacks accuracy; nevertheless, their regulation in regard to the Se exposure investigated in this study and other stressors (Gregorc et al., 2018a) remains a strong evidence of their involvement in the honey bee detoxification process.

From a field perspective exposure, a direct correlation between Se levels in plants (corn and soybean) and Se levels in soil was previously described (Sun et al., 1985). In highly Se rich soils, corn and soybean could average 8.1 and 11.9 μg Se g^−1^ respectively (Yang et al., 1983). Some other plant species such as *Brassica juncea* and *Stanleya pinnata* (Brassicaceae) could typically accumulate 5000 mg Se kg^−1^ DW in the field (Galeas et al., 2008) (Table 3), nonetheless, it is not clear how much Se of these concentrations are available for bees in both nectar and pollen. A previous study demonstrated significant accumulation of Se in *S. pinnata*’s pollen (12,900 μg/g DW) and nectar (150 μg/g WW) when irrigated with 8 μM selenate (Hladun et al., 2011). It should also be noted that 14 mg Se kg^−1^ was identified in pollen (Table 3) collected from bees foraging on plants growing in coal-fly-ash environment in New York, while no Se was detected in pollen from a provenance of plants growing on field soil (De Jong et al., 1977). Further investigation is needed for a larger range of plants pollinated by bees, on the precise Se levels available for bees in nectar, pollen and guttation.

In conclusion, our study shows that honey bees are vulnerable to slight augmentation of Se in their diet (0.6–6) μg Se mL^−1^ and significant bee mortality is recorded at 6 μg Se mL^−1^. The highest accumulation of Se residue that living bees can tolerate is 0.12 μg Se bee^−1^. Selenium induces notable oxidative stress in honey bees leading to protein damage at high concentrations. In an effort to alleviate oxidative stress, honey bees upregulated considerable sets of antioxidant genes, including uncharacterized “selenoprotein genes”, more particularly a Selenium binding protein gene required for the translation of selenoproteins mRNA. Finally, our study was conducted on caged adult bees, and results may vary in the context of hive environment or other honey bee developmental stages as shown in previous studies. It should also be noted that bee behavioral disruptions caused by Se sublethal doses might be as important as direct toxicity.

## Figures and Tables

**Table 1:** Honey bee uncharacterized selenoprotein-like genes and Selenium binding protein investigated in this study. Available gene characteristics are provided along with primer sequences and accession numbers.

Characteristic	SELENOT	SELENOK	SELENOF	Sbp2
Gene Description	Thioredoxin reductase-like selenoproteins T homolog	Selenoprotein K-like	Selenoprotein F	Selenium binding protein 2
Accession	XM_623426.6	NM_001278332.1	XM_006557387.3	XM_001122154.5
Locus	LOC550992	LOC551304	LOC410663	LOC726417
Gene length nt	1390	804	980	2407
N of exon	4	4	4	6
Primer F	GACAGCCACCAGCATCATTG	TGGAAGCGTTTTATGTGGTACT	GCTGATGATTGTAAAAGTTTAGGCT	AGGTTTGAACGATGCTCTCG
Primer R	TGGACCACACAGGAACATCAT	ATCTACGAGTTGGTGGACGTG	TTCGAGAACAGCACGTGGAT	AGGGGCGATAATCACGAGTT
Amplicon Length bp	150	182	174	172

**Table 2:** Major Honey bee antioxidant genes investigated through RT-qPCR in this study. Target and housekeeping genes, primer sequences are provided along with their accession numbers and amplicon size.

Gene Code	Gene Description	Accession Number: NCBI/Beebase	Primer F and R	Amplicon Size bp
*1 - TARGET GENE*				
Cat	Catalase	NM_001178069.1	ACGAAATCCTTCCGCTGACC AGCATGGACTACACGTTCCG	211
Trxr1	Thioredoxin Reductase	AY329357.1	GCAAGTACTGTTGCCCAGGA GTGTTTGTCTATCTTTATCCACCCA	130
Sod1	Superoxide dismutase 1	NM_001178027.1	CGTTCCGTGTAGTCGAGAAAT GGTACTCTCCGGTTGTTCAAA	101
MsrA	Methionine sulphoxide reductase A	NM_001178047.1	GGGCCGGTGATTGTTTATTTG CAACGACTTCTGTATGATCACCT	120
*2 - HOUSEKEEPING GENES*				
GAPDH	Glyceraldehyde 3-Phosphate Dehydrogenase	XM_393605.6	CTGCACAGACCCGAGTGAAT CCGAACTCAATGGAAGCCCT	105
RPS18	40S Ribosomal Protein S18	XM 625101.6	AGCGTGCTGGAGAATGTTCA CCACGTACACGTAAACCCCA	238

**Table 3:** Summary of Selenium concentrations previously found in various plants, soils, pollen and nectar. Selenium found in hive products such as honey, trapped pollen and foragers are also given along with the residues of Se in the dead bees found in our current study.

Component	Se concentration mg/kg	Potential Bee Exposure	Location	Reference
*Honey bee:*				
Dead bees fed (0.6 μg Se mL^−1^)	0.1	Bee body	Lab experiment - USA	Current study
Dead bees fed (6 μg Se mL^−1^)	0.91	Bee body	Lab experiment - USA	Current study
Dead bees fed (60 μg Se mL^−1^)	6.02	Bee body	Lab experiment - USA	Current study
Dead bees fed (600 μg Se mL^−1^)	10.29	Bee body	Lab experiment - USA	Current study
Foragers	7.03	Bee body	Urban area - Poland	Roman (2010)
Foragers on High-Se flowers	13.9–15.7	Bee body + pollen	Fort Collins, CO, USA	Quinn et al. (2011)
*Plant:*				
Astragalus, Xylorrhiza, Stanleya, Oonopsis	3000–10,000	Guttation, nectar, pollen	West of USA	Freeman et al. (2006) and Spallholz (1994)
Native plants	1000	Guttation, nectar, pollen	WY - USA	Spallholz (1994)
Plant in normal soil	< 3	Guttation, nectar, pollen	Anywhere	Whanger (2002)
Brassicacea, Asteraceae, Fabaceae	1000	Guttation, nectar, pollen	Anywhere	Galeas et al. (2008); Terry et al. (2000)
Nectar *S. pinnata* DW/FW	2323/275	Direct	Fort Collins, CO, USA	Quinn et al. (2011)
*Soil:*				
High Se content	4–5	Dust, plant uptake	Northern Great Plains of USA	Rosenfeld and Beath (1964)
Average Se	3	Dust, plant uptake	European Union	Kabata-Pendias and Adriano (1995)
Range of Se	0–1500	Dust, plant uptake	Ireland	Oldfield (1999)
*Bee Hive Product:*				
Honey	0.73	Product	Seleniferous areas USA	Quinn et al. (2011)
Honey	0.03 – 0.11	Product	Turkey	Tuzen et al. (2007)
Honey FW	0.4–1.0	Product	CO - USA	Quinn et al. (2011)
Trapped Pollen	14	Product	Fly ash-areas	De Jong et al. (1977)
*Other Organism:*				
Rat/Chronic Se toxicity	3–16	Body	Lab experiment	Brasher and Ogle (1993) and Wilber (1980)
Bumble bees on High-Se flowers	228–274	Body + pollen	Fort Collins, CO, USA	Quinn et al. (2011)
